# Causal Effect of Honorary Titles on Physicians’ Service Volumes in Online Health Communities: Retrospective Study

**DOI:** 10.2196/18527

**Published:** 2020-07-09

**Authors:** Haiyan Yu, Yali Wang, Jying-Nan Wang, Ya-Ling Chiu, Hang Qiu, Mingyue Gao

**Affiliations:** 1 Chongqing Key Laboratory of E-commerce and Modern Logistics Chongqing University of Posts and Telecommunications Chongqing China; 2 College of International Finance and Trade Zhejiang Yuexiu University of Foreign Languages Shaoxing China; 3 College of International Business Zhejiang Yuexiu University of Foreign Languages Shaoxing China; 4 School of Computer Science and Engineering University of Electronic Science and Technology of China Chengdu China; 5 Life Course Epidemiology and Biostatistics, Population, Policy, and Practice Programme Great Ormond Street Institute of Child Health, University College London London United Kingdom

**Keywords:** causality, health information systems, organizational policy, physician-patient relations, remote consultation

## Abstract

**Background:**

An OHC online health community (OHC) is an interactive platform for virtual communication between patients and physicians. Patients can typically search, seek, and share their experience and rate physicians, who may be involved in giving advice. Some OHC providers provide incentives in form of honorary titles to encourage the web-based involvement of physicians, but it is unclear whether the award of honorary titles has an impact on their consultation volume in an OHC.

**Objective:**

This study is designed to identify the differential treatment effect of the incentive policy on the service volumes for the subgroups of treatment and control in an OHC. This study aims to answer the following questions: Does an honorary title for physicians impact their service volumes in an OHC? During the period of discontinuity, can we identify the sharp effect of the incentive award on the outcomes of physicians’ service volumes?

**Methods:**

We acquired the targeted samples based on treatment, namely, physicians with an honorary title or not and outcomes measured before and after the award of the 2 subgroups. A regression discontinuity design was applied to investigate the impact of the honorary titles incentive as a treatment in an OHC. There was a sharply discontinuous effect of treatment on physicians’ online health service performance. The experimental data set consisted of 346 physicians in the treatment group (with honorary titles). Applying the propensity score matching method, the same size of physicians (n=346) was matched and selected as the control group.

**Results:**

A sharp discontinuity was found at the time of the physician receiving the honorary title. The results showed that the parametric estimates of the coefficient were significantly positively (*P*<.001) associated with monthly home page views. The jump in the monthly volumes of home page views was much sharper than that of the monthly consultations.

**Conclusions:**

The changes in the volumes of monthly consultations and home page views reflect the differential treatment effect of honorary titles on physicians’ service volumes. The effect of the incentive policy with honorary titles is objectively estimated from both the perspective of online and offline medical services in an OHC. Being named with honorary titles significantly multiplied monthly home page views, yet it did not significantly impact monthly consultations. This may be because consultation capacity is limited by the physician's schedule for consultations.

## Introduction

### Background

Online health communities (OHCs) are an essential channel for creatively allocating health care resources among widely distributed patients in modern life. An OHC is usually referred to as an interactive platform between patients and physicians for virtual communication. Patients can search, seek, and share their experience of medical advice and rate the physician as social returns in an OHC. Meanwhile, physicians can share their medical knowledge and provide online medical services. An OHC is a convenient, real-time supplement for physician-patient interaction (PPI) without limitations on time and space [1]. Physicians’ web-based involvement (ie, the service volumes) is a determinant factor of OHCs’ success in health service delivery [2]. The platform management provider of an OHC often provides incentive policies (ie, honorary titles) to encourage physicians’ web-based involvement. For example, 346 physicians on the *Good Doctor* platform were awarded the honorary title *2017 Annual Good Doctor* in January 2018. According to a survey of large OHC firms by the Towers Watson/National Business Group on Health [3], 69% reported that they offered wellness incentives and that the size of the incentives increased with time. However, it is still unclear whether the award of honorary titles has an impact on their consultation volume in an OHC.

With the quick development of OHCs, the physician-patient online interaction has gained more attention from scholars all over the world, especially regarding what incentive mechanisms and strategies can be designed to prompt sustained PPIs in OHCs [4]. In the bidirectional process of PPIs [5], patients seek information and make the decision to select a physician for consultation, and then, the physicians share their medical knowledge and provide medical services. Patients could also provide returns (rate, vote, and share experience online) for physicians. After receiving feedback and returns, physicians could balance their efforts for the subsequent PPI process. A good PPI can benefit both patients and physicians. It can provide patients with a truly information-based selection process and good outcome of the consultation process. In addition, a good PPI can provide physicians with returns that can also affect their reputation. Incentive policies have been widely developed to encourage PPI [3]. The high rating or award of good doctors online is thought to be a good indicator. This indicator not only represents the praise of the physician but can also predict the following process of PPI theoretically. Recently, there have been more studies focusing on the impact of incentive policies (high rating or honorary title of *Good Doctor*) in OHCs [1]. A previous study reported that physicians’ online contributions and reputation were closely associated with patients’ decision-making process when seeking medical consultation [6]. Due to the cross-sectional study design, the conclusion can only be explained in terms of association [7]. To evaluate the causal impact of incentive policy on physicians’ consultation volumes, a sequential occurrence needs to be considered and analyzed in causal design, as appropriate.

In this study, we intend to estimate the causal effect of an incentive strategy on the PPI process of an OHC. Our analysis uses the information from the biggest OHC in China (*Good Doctor* website) [1], which provides reliable information on more than 10,000 hospitals and more than 640,000 physicians across the country. The specific treatment in our study is the award of the honorary title of *Annual Good Doctor*, which is assigned by a threshold of a weighted score summarizing the hospital level, the professional capital, number of votes, and the experience of health care service of patients. The dimensions of treatment included both temporal (pre- and post-treatment) and treatment (treated and untreated group) effects. The outcome contained 2 aspects [8]: the home page view frequency and the online consultation frequency. The home page view frequency mainly represents the physicians’ reputation and effort from the patients’ side. The online consultation frequency further includes the accessibility and efforts of physicians when chosen for online services. The 2 indicators together represent the performance of the continuous PPI process in OHCs.

### Literature Review

Although the natural experiment design is widely adopted to evaluate the policy’s causal effects in empirical research [9], the experiment remains difficult or impossible to implement because of ethical, political, and financial reasons. A large share of the empirical work on policy evaluation relies on observational data, in which policies are determined in a way other than through randomization assignment. Drawing the inference of a policy’s causal effect based on observational data is quite challenging, especially for incentive policy evaluation in an OHC setting. Several methodological issues need to be overcome by adopting causal methods creatively because of the various weaknesses of classic methods.

The counterfactual impact of the treatment needs to be estimated in causal inference. *Treatment* is a general term referring to certain interventions of interest, for example, the incentive policy in our study. An important prerequisite is that the treated and untreated groups are comparable and balanced to draw unbiased causal effects, which can be achieved by randomization in an experimental setting. However, in an observational study, treated and untreated groups may differ in observed and unobserved characteristics, which can affect the assignment of treatment and outcome. Several techniques such as multiple linear models and extensions, widely adopted in previous studies to identify the association between rewards and physicians’ contribution [1], only control for observed confounders. Propensity score matching (PSM) [10,11] mimics the randomization process, reduces the confounding on the treatment assignment, and reaches a balanced group sample (with simulated counterfactual control units) in an observational setting. With the propensity score defined [12] as a conditional probability of assignment to treatment based on covariates, PSM largely reduces the matching process from multiple dimensions to a single dimension [13]. The matching process is also appropriate for temporal case matching [14] and the classification of medical cases [15].

To mimic the experimental design with observational data, both *treatment* and temporal effects need to be considered. With the treated samples, a simple comparison of outcomes between pre- and post-treatment could be contaminated by the effects of other events (except the treatment) that occurred during both periods. For example, the seasonable factor may affect the change in the outcomes of OHCs [1]. With both treated and control groups, the comparison of post-treatment outcomes could capture more than the treatment effect, even after controlling for observed confounding [16]. The reason lies in the difference in unobserved attributes between the treated and untreated groups. Difference-in-differences (DID) [17], combined with PSM to achieve a parallel trend assumption (2 groups would show a parallel trend if neither of them experienced the treatment effect), is a useful method to reduce the impact of extraneous factors and selection bias. DID compares the average change over time in the outcome of the treated group with that of the control (untreated) group [18].

In many practical cases, the treatment assignment is (partially) determined by a cutoff or a threshold. This advanced design, known as a sharp regression discontinuity design (RDD) [19], is an extension of DID. Despite comparing the observations of the pre- and post-treated outcomes available in both groups, RDD also shows a good capacity for causal inference when the treatment assignment is deterministic and discontinuous at the cutoff. Comparing the observations close to the cutoff (local treatment effect) would achieve local *randomization* near to the threshold.

This study aims to estimate the causal effect of an incentive strategy on the PPI process of an OHC. In accordance with the previous discussion on methodology, PSs were estimated as the predicted probabilities of treatment (being awarded honorary titles based on covariates). Score matching was then conducted to reach a comparable control sample with the treatment group. Considering both the temporal and treatment dimensions of the policy, the DID idea was applied to compare the average pre- and posttreatment changes in the treated group (with the honorary title) with those in the untreated group. If the relationship between the covariates and the potential outcomes is *smooth* around the threshold (in covariates), the discontinuity (sharp jump) created by the treatment can provide local randomization. RDD would then be implemented appropriately to evaluate the causal effect of treatment (receiving the honorary title) on both outcomes (home page view and online consultation frequency) at the threshold.

In summary, this study aimed to identify the average change in home page views and online consultation volume for physicians with the honorary title versus those without the honorary title. The investigation attempts to answer the following questions: (1) Does there exist the average treatment effect (ATE) of the honorary title on changes in outcomes (physicians’ home page views and consultation volumes) in an OHC? (2) At the discontinuity of the *treatment* assignment, does the sharp effect of the incentive award exist on the outcomes, and can it be identified?

## Methods

### Research Models

**Figure 1:**
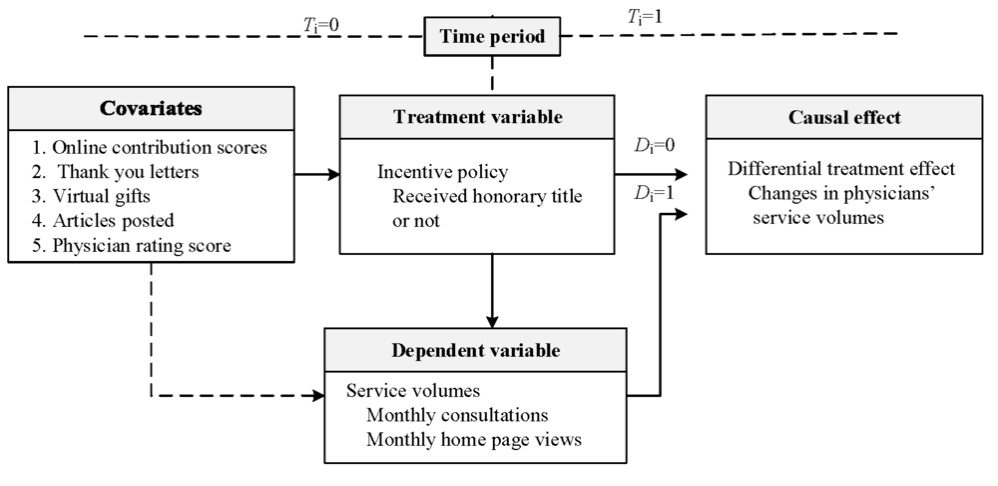
Framework of the causal effect study on the outcome of physicians’ service volumes, with D=1 indicating that physicians received honorary titles (in the treatment group), D=0 indicating the control group, T=1 indicating the postaward period, and T=0 indicating the preaward period.

This section demonstrates the research framework of this study. To investigate the differential treatment effect, the incentive policy was regarded as the treatment in the research design. The research framework was demonstrated to investigate the differential treatment effect of the incentive policy on physicians’ service volumes, as illustrated in Figure 1. This service volume also reflected the patients’ choice of physicians online.

First, 5 covariates were considered in this observational study. These variables are the physician rating score (PRS), the number of thank you letters, the number of virtual gifts, the number of online contribution scores, and the number of articles posted. The covariates represent the doctor’s characteristics at a specific time point. Second, the honorary titles of the physician awarded from the OHC were viewed as the treatment. Within the causal inference mechanism, the objects of interest were those physicians assigned as the award recipient or not (*D_i_*). This time factor helped in distinguishing the factors of the cause and effect among those variables. Third, to further investigate the dynamism of the effect on the changes in their service volumes, the time periods (*T_i_*) were divided into those before awarding and after awarding. Fourth, the service volume is measured by 2 factors: the number of monthly consultations and the number of monthly home page reviews. These 2 factors reflect the online service (home page reviews) and the offline service (serviced patients of medical consultations), respectively. The number of patients that physicians serviced monthly (Patients #) and the number of their home page views monthly (Views #) [20] can be viewed as the proxies of the outcomes. Thus, these 2 proxy variables were set as the dependents. Moreover, the initial states of the numbers of those 5 covariates are measured as their cumulative before the examined period (June 25, 2017).

The definitions and measurements of all variables are presented in Table 1. *Patient_i_ (t)* is measured as the number of online consultations (for patients) provided by the physician *i* in month *t*. *Views_i_ (t)* is measured as the number of online home page views of the physician *i* in month *t*. The covariates were considered for case-control matching. *PRS_i_* is measured as the PRS (by patients), which refers to the star scores listed on the OHC website. *Thank_i_ (t)* is measured as the mean of the number of thank you letters of the physicians. *Gift_i_ (t)* is measured as the mean of the number of gifts received by the physician *i* in month *t*. *Contr_i_ (t)* is measured as the mean of the contribution score of physician *i* in the month *t*, which refers to the contribution scores listed on the website. *Aritcle_i_ (t)* is measured as the mean of the number of physician articles. *N* is the number of physicians in the experimental data.

**Table 1:** Variable definitions and measurements.

Variables		Definitions	Measurements
**Causal effect**			
	*RDDeffect*	Differential treatment effect	The causal effects of honorary titles incentives (treatment) in OHC^a^ with RDD^b^
**Dependent variable Y**			
	*Patient*_i_ *(t)*	Number of monthly consultations	Number of online consultations (for patients) provided by the physician *i* in the month *t*
	*Views*_i_ *(t)*	Number of monthly home page views	Number of online home page views of the physician *i* in the month *t*
**Treatment variable**			
	*D*_i_	Receive honorary title or not	*D*_i_*=1* indicates physician *i* was titled as *2017 Good Doctor* in OHC, otherwise *D*_i_*=0*
**Time periods**			
	*T*_i_	Preaward or postaward period	*T*_i_*=0* indicating the period before January 2018 (date of the honorary title), otherwise *T*_i_*=0*
**Covariates**			
	*Contr*_i_ *(t)*	Contribution score	Total online contribution score calculated by OHC for physician *i* in the month *t*
	*Thank*_i_ *(t)*	Number of thank you letters	Total number of online thank you letters physician *i* received in the month *t*
	*Gift*_i_ *(t)*	Number of virtual gifts	Total number of online virtual gifts physician *i* received in the month *t*
	*Article*_i_ *(t)*	Number of articles posted	Number of articles posted by physician *i* in the month *t*
	*PRS_i_ (t)*	PRS^c^	Mean of the rating scores by patients for physician *i* in the month *t*

^a^OHC: online health community.

^b^RDD: regression discontinuity design effect.

^c^PRS: physician rating score.

### Data Collection

This study used existing records to conduct a retrospective study. The requirement for individual doctor consent was waived as the study did not impact clinical care and all data were deidentified. None of the data collected for the study were related to private information about the physicians.

**Figure 2:**
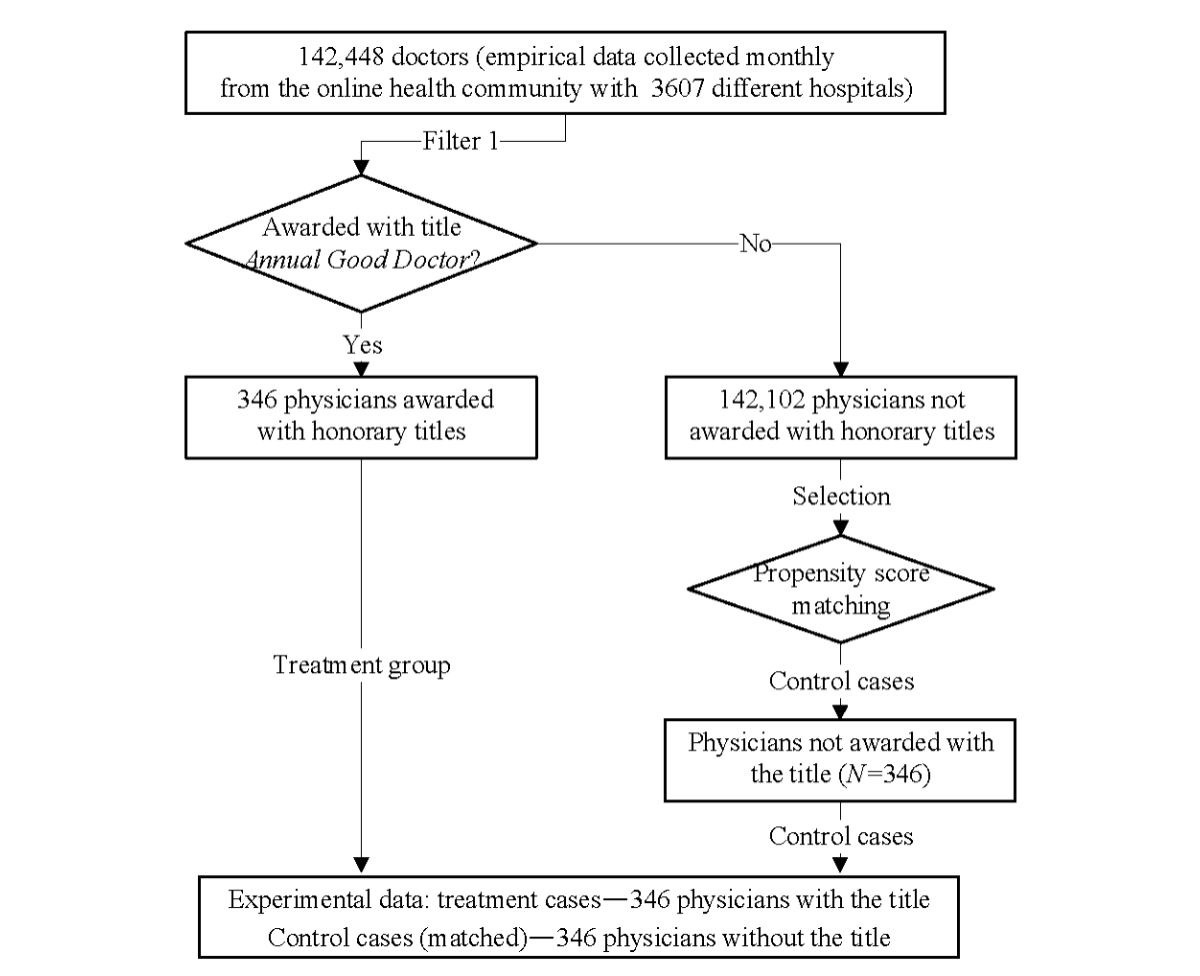
Data collection and preprocessing procedures.

Through the web crawler technology, a longitude data set from July 26, 2017, to June 26, 2018, was collected and filtered monthly from the Good Doctor website for the study. The collected data set contained the variables used in this study and other deidentified information. Among the online physicians, over 140,000 were involved and participated in OHC [1] with their personal profiles (including personal home pages). In January 2018, 346 of them were awarded the honorary title *2017 Annual Good Doctor.* Honorary titles were rated based on the number of consultations, the number of appointment referrals (patients), patient satisfaction with online services (the review rating score), and other factors. The flowchart of data acquisition and filtering process is shown in Figure 2.

**Figure 3:**
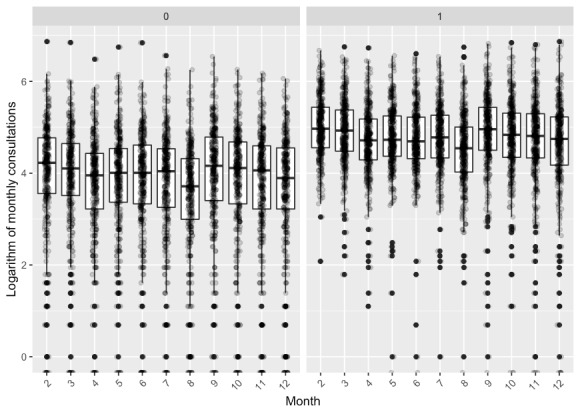
Distribution of the logarithm of monthly consultations, with 0 indicating the control group and 1 indicating the treatment group.

**Figure 4:**
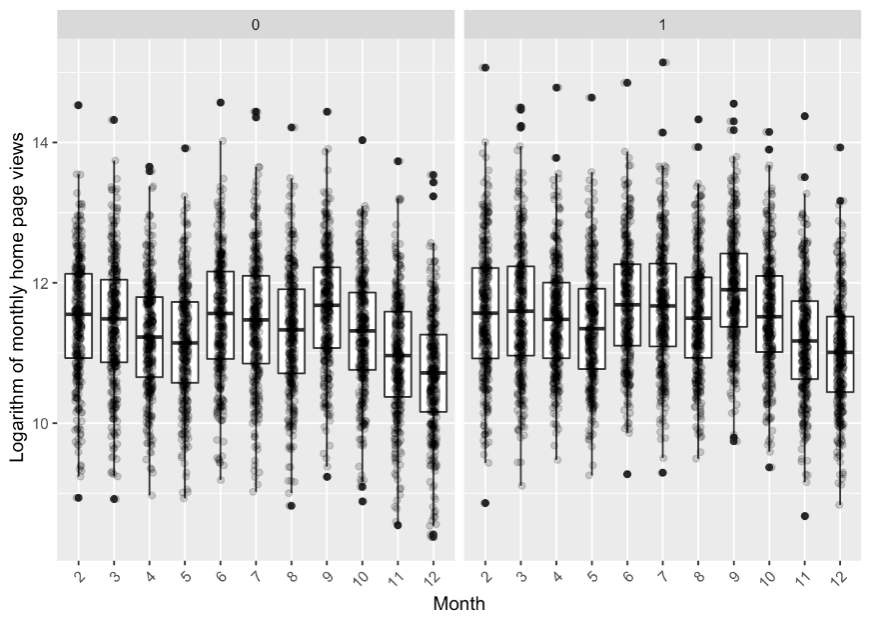
Distribution of the logarithm of monthly home page views, with 0 indicating the control group and 1 indicating the treatment group.

During the preprocessing, outliers were removed from the original data. The design of the study is based on the propensity score matching (PSM) with 1:1 matching. The distribution of the logarithm of monthly consultations and that of the monthly home page views are illustrated in Figures 3 and 4. In these figures, 0 indicates the control group and 1 indicates the treatment group. The distribution results suggest that the causal effect cannot be estimated with the distributions directly.

**Estimation of RDD Effect**

Through the RDD model, this study attempted to identify the ATE [21] of honorary titles for physicians on the changes in their service volumes. The theoretical contributions of this study not only lie in the design for estimating the RDD effect but also in combining it with the DID model through prediction of the counterfactual outcomes of the matching samples. The prediction of the RDD effect can be modeled as [22] follows:


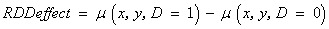


Where 
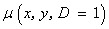
 and 
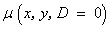
 are the estimated expectation of the treatment group and the control group with the covariates *x* (ie, time period) and the outcomes *y* (ie, *Patient_i_ (t)* and *Views_i_ (t)*), respectively.

To derive a balanced control-treatment case data set, we trained the logistic regression to estimate the PS [23].


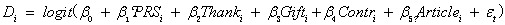


where *β_0_* is the coefficient of the constant term and *β_j_*, *j*=1,..., 5, are the coefficients of the covariates, as detailed in Table 1. The error term 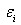 obeys a normal distribution with mean and variance 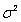.

We then matched control-treatment cases on pretreatment covariates with the PS. In the matching process, the scalar (N) can be preset for the number of matches needed (ie, the default value 1 is for one-to-one matching). More similar units are more likely to experience similar trends to meet the parallel path assumptions. Thus, the bias of the RDD effect can be reduced with such data sets of control-treatment pairs.

## Results

In this section, we describe the results with descriptive statistics, the overlap assessment, and the differential treatment effect of honorary titles. Our findings provide empirical evidence that a regression discontinuity exists at the cutoff of the period.

### Descriptive Statistics

The statistics of the empirical experimental data for each month are shown in Table 2. The state column illustrates the initial mean value of the experimental period, and the following 11 columns show the marginal changes per month. For example, PRS in August 2017 was 0.01, which means that it increased by 0.01 on average from the mean value (4.58) in the last month (July 2017). As the certification date of honorary titles (award) was January 20, 2018, the examined periods of treatment were from January 2018 to June 2018 (6 months).

From Table 2, the results show that for D=1 (award), monthly changes of preaward PRS were positive, whereas for D=0 (nonaward), there were negative changes. In terms of the thank you letters, the monthly data of D=1 (award) was higher than that of D=0 (nonaward). Similar trends were observed for virtual gifts, posted articles, contribution scores, consulted patients, and home page views.

**Table 2:** Statistics of the empirical experimental data.

Variables		State^a^	T=0 (Monthly data^b^)					T=1 (Monthly data)						
		July 2017	August 2017	September 2017	October 2017	November 2017	December 2017		January 2018	February 2018	March 2018	April 2018	May 2018	June 2018
**D=1**														
	PRS^c^	4.58	0.01	0.008	0.008	0.007	0.002		0.006	–0.003	–0.003	0.002	–0.005	0.002
	Thank^d^	139.3	6.6	6.4	5.8	4.8	5		5.4	4.1	4.4	5	6.2	6.8
	Gift	711.4	25.4	22.2	26.1	20.7	17.3		19.6	19.6	18.1	16.6	14.6	14.2
	Contr^e^	87370	3699	3684	3337	3464	3557		3823	3187	4351	4157	3960	3946
	Article	63.77	1.01	1.19	1.05	0.87	1.07		1.42	1.49	1.29	1.05	1.57	0.89
	Patient#^f^	5771	186	173	143	149	150		151	123	182	164	157	148
	Views#^g^	4,099,352	173,977	184,098	145,770	128,537	180,300		181,598	148,503	214,388	153,022	105,504	86348
**D=0**														
	PRS	4.493	–0.007	–0.005	–0.004	–0.001	–0.009		–0.003	–0.014	–0.009	–0.007	0.001	–0.02
	Thank	121.8	4.2	4.1	3.3	2.8	3		3	2.4	2.4	0.5	5.98	3.62
	Gift	611.5	14	10.9	14.8	10.3	9.2		9.6	9.9	8.6	8.2	8.1	6.9
	Contr	78281	1515	1471	1331	1436	1475		1475	1200	1613	1457	1352	1500
	Article	50.57	–2.02	0.61	0.52	0.45	0.55		0.47	0.37	0.44	0.65	0.2	0.31
	Patient#^f^	5220	87	79	65	74	72		70	54	83	43	77	65
	Views#^g^	4,882,225	143,432	141,268	107,423	101,340	151,260		153,008	120,576	164,884	30,649	115,264	65,165

^a^Initial state of the recorded data.

^b^Monthly change of the recorded data.

^c^PRS: physician rating score.

^d^Thank: Thank you letters.

^e^Contr: contribution score.

^f^Mean of the monthly consultations of the group.

^g^Mean of the monthly home page views of the group.

### Overlap Assessment

The first step in analyzing the experimental data was to estimate the PSs using a logistic regression model with one main effect (on treatment) for each covariate. In the estimation of PS, the dispersion parameters for the binomial family were taken to be 1. With many covariates, it is difficult to examine the numeric diagnostics carefully for each covariate. As usual [24,25], graphical diagnostics are helpful for quickly assessing the covariate balance. Although the densities of raw treated and matched treated cases did not change, those of raw controls and matched controls changed significantly. The absolute standardized difference is defined as follows [26]:


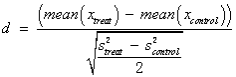


where *mean (x_treat_)* and *mean (x_control_)* denote the sample mean of the covariate in treated and control units (physicians), respectively, and *s^2^_treat_* and *s^2^_control_* denote the sample variance of the covariate in treated and control units, respectively.

**Figure 5:**
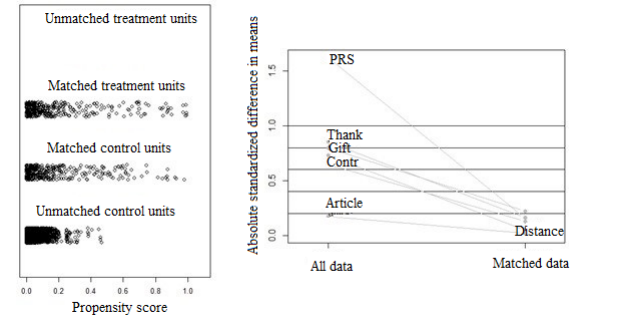
Distribution of propensity scores with experimental data. The left subfigure illustrates the distribution of propensity scores, and the right subfigure illustrates the absolute standardized difference in means of all data and matched data. Contr: Contribution score; PRS: physician rating score.

Figure 5 shows the weighted dots by their proportional size, which is also useful for stratification. Meanwhile, the absolute standardized difference is helpful for comparing the mean of continuous variables between the 2 groups, as illustrated in Figure 5 (right). The results show an adequate overlap of the PSs, with a good control match for each treated unit.

### Differential Treatment Effect of Honorary Titles

**Figure 6:**
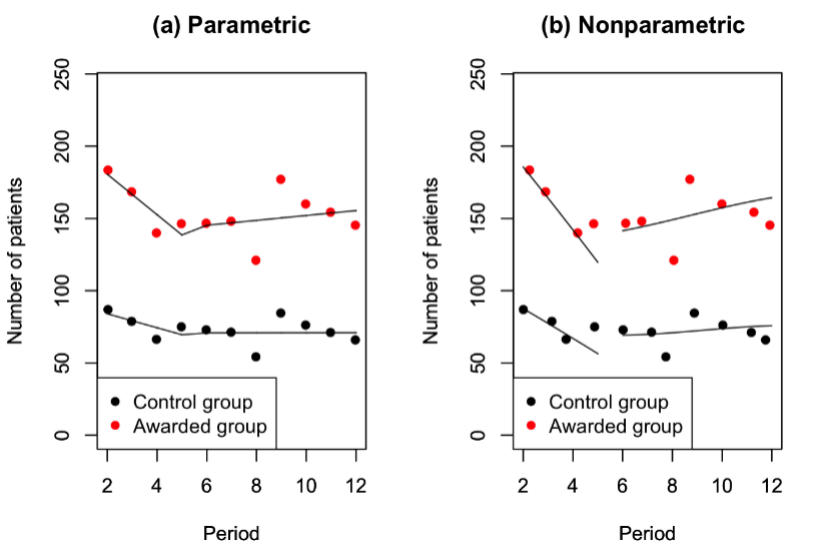
Estimation of the number of monthly consultations before and after physicians receive honorary titles.

**Figure 7:**
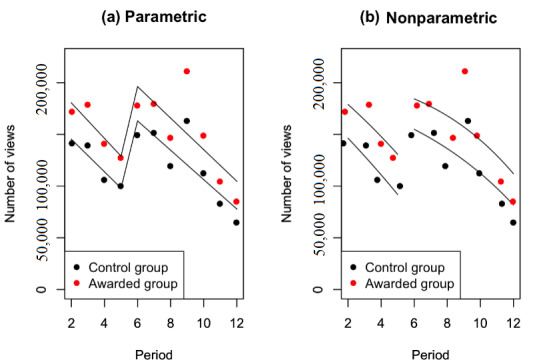
Estimation of the number of monthly home page views before and after physicians receive honorary titles.

This treatment effect illustrates the impact of honorary titles on the changes in physicians’ service volumes. Two tests for the estimation of the impact of honorary titles were carried out with the panel data of 12 months. The period of control was investigated from July 2017 to December 2017, and the period of treatment was from January 2018 to June 2018. Among them, the first month of control was used to acquire the initial state of the system. The marginal quantity of the sequential periods was then acquired accordingly. The impacts of honorary titles on monthly consultations and home page views were analyzed using the panel data, as demonstrated in Table 3. The estimation of the number of monthly consultations before and after doctors receiving honorary titles is also visually illustrated in Figure 6. Similarly, this study also investigated the estimates of the number of monthly home page views, and the results are shown in Figure 7.

**Table 3:** Parametric and nonparametric estimates of the coefficient (the treatment effect).

Group and methods			Estimate	SE	N^a^	*P* value
**Monthly consultations**						
	**Control**					
		Parametric	6.113	5.970	3696	.31
		Nonparametric	–9.286	8.087	1680	.25
	**Treatment**					
		Parametric	20.699	9.095	3806	.02
		Nonparametric	5.133	10.277	2422	.62
**Monthly home page views**						
	**Control**					
		Parametric	80,666	10,704	3696	<.001
		Nonparametric	66,814	13,535	2352	<.001
	**Treatment**					
		Parametric	84,340	14,535	3806	<.001
		Nonparametric	73,351	18,386	2422	<.001

^a^N: number of physicians in the experimental data.

In Table 3, the results show that the parametric estimates of the coefficient (the treatment effect of the honorary title) are significantly positive (*P*<.001) on monthly home page views. The estimates were 80,666 for the control group and 84,340 for the treatment group. Similar results were obtained for the nonparametric estimates of the coefficients, which were 66,814 for the control group and 73,351 for the treatment. These results indicate that the physicians with honorary titles had more monthly home page views than their counterparts. Meanwhile, honorary titles highlighted the physicians, accelerating the increase of the monthly home page views more greatly than the others. The number of monthly consultations of the physicians with the honorary titles was larger than those without the titles.

However, the parametric estimates of the effect on monthly consultations are positive (6.113 for the control and 20.699 for the treatment group) but not significant. The nonparametric estimates of the effect on monthly consultations were negative (–9.286) for the control and positive (5.133) for the treatment group (also insignificant). These results support our argument that the effect of honorary titles for physicians can significantly multiply the increases in the monthly home page views, yet they cannot significantly impact the monthly consultations.

In Figure 6, despite the decreasing treads, there was also a jump for monthly consultations during the period of receiving honorary titles. The results also indicated that 2 months of lag existed in the RDD. However, the jump in the volume of monthly consultations was insignificant or did not occur instantly. The honorary titles of physicians were awarded in January 2018, whereas a sharp regression discontinuity occurred in March 2018.

In Figure 7, the results show that honorary titles caused a jump in the monthly volume of home page views, that is, a sharp regression discontinuity. Moreover, the trends of the period before the jump were decreasing, which illustrated that the counterfactual observations of monthly volumes would be much less than those before this jump. Therefore, these results provide empirical evidence that regression discontinuity existed at the cutoff of the period.

## Discussion

### Principal Findings

In this paper, we investigated the causal effect of honorary titles for physicians in terms of the changes in their service volumes in online health care communities. Monthly home page views and consultations were chosen as the 2 proxy variables for the outcomes. To identify the causal effect, multiple covariates, including physicians’ rating scores, thank you letters, virtual gifts, online contribution scores, and posted articles, were considered for PS estimation. With pure randomization, the bias of effect estimation was reduced with the assigned samples. Through PSM, the results showed an adequate overlap of the PSs, with a good control match for each treated case. The results showed that honorary titles caused a jump in the monthly volumes of monthly consultations and home page views, specifically a sharp regression discontinuity.

Compared with the discontinuity regressions, the jump in monthly consultations is not as sharp as that of home page views. There may be many reasons for these sharp regression discontinuities. For example, if honorary titles are implemented for ranking and deploying physicians to users, then entitled physicians get more clicks because they appear first. However, consultation capacity is limited by the physician’s schedule. This leads to the limit of the increase in monthly consultations for physicians. In contrast, there is no limit to the increase in home page views, which causes the jump in the monthly volumes of home page views to be much sharper than that of the monthly consultations. In total, changes in the monthly volumes of monthly consultations and home page views reflect the differential treatment effect of honorary titles on physicians’ service volumes. The effect of the incentive policy with honorary titles is objectively estimated from the perspective of both online and offline medical services.

Although the causal study design was performed rigorously with the PSM method, this study still has some limitations. The number of articles posted by physicians was collected monthly for this study, with potential seasonable noise data. Meanwhile, the historical monthly data of the home page views and the online consultation frequency may be a cause of the honorary title for physicians in the current period. In this study, we introduced the time factor to distinguish the factors of the cause and effect among these variables. In future studies, their historical data can also be implemented as covariates to improve the balance of the comparing groups in the causal inference. To further investigate the proxy of physicians’ service volumes, more characteristics could be abstracted from physicians’ articles. In addition, falsification checks could also be implemented in future studies, including investigating whether covariates jump during the period of honorary titles (when jumps occur at cutoff thresholds) and what are the most reasonable time lags.

### Conclusions

In OHCs, the platform management provider offers incentive policies (eg, honorary title) to encourage physicians’ web-based involvement. However, the impact of the incentive policy on patients’ online choice of physicians is still unclear. In this study, we investigated the causal effect of honorary titles for physicians on changes in their service volumes, including monthly home page views and consultations. By stratifying the samples with the treatment (honorary title) and the period (pre- vs postaward), the RDD method was applied to identify the impact of the incentive policy on the service volumes of physicians. A sharp discontinuity was found at the time of the physician receiving the honorary title. The results showed that both parametric and nonparametric estimated coefficients were significantly positive (*P*<.001) for monthly home page views. The effect of honorary titles for physicians can significantly multiply the increases in the monthly home page views, yet its impact on monthly consultations was insignificant. Therefore, these results provide empirical evidence for our claim that regression discontinuity existed at the cutoff of the period. In the future, more investigations can be conducted to identify the time lag of the RDD effect.

## Abbreviations

ATE: average treatment effect
DID: difference-in-differences
OHC: online health community
PPI: physician-patient interaction
PRS: physician rating score
PSM: propensity score matching
RDD: regression discontinuity design
